# Imaging-based characterization of convective tissue properties

**DOI:** 10.1080/02656736.2020.1845403

**Published:** 2020-12

**Authors:** D. Fuentes, E. Thompson, M. Jacobsen, A. Colleen Crouch, R. R. Layman, B. Riviere, E. Cressman

**Affiliations:** a Departments of Imaging Physics, The University of Texas M.D. Anderson Cancer Center, Houston, TX, USA;; b Department of Computational and Applied Mathematics, Rice University, Houston, TX, USA;; c Interventional Radiology, The University of Texas M.D. Anderson Cancer Center, Houston, TX, USA

**Keywords:** Modeling (i.e., heat transfer, ultrasound, EM, integrated, treatment planning), mass transport, mixture theory, Darcy law

## Abstract

Convective transport is an important phenomenon for nanomedicine delivery. We present an imaging-based approach to recover tissue properties that are significant in the accumulation of nanoparticles delivered *via* systemic methods. The classical pharmacokinetic analysis develops governing equations for the particle transport from a first principle mass balance. Fundamentally, the governing equations for compartmental mass balance represent a spatially invariant mass transport between compartments and do not capture spatially variant convection phenomena. Further, the parameters recovered from this approach do not necessarily have direct meaning with respect to the governing equations for convective transport. In our approach, a framework is presented for directly measuring permeability in the sense of Darcy flow through porous tissue. Measurements from our approach are compared to an extended Tofts model as a control. We demonstrate that a pixel-wise iterative clustering algorithm may be applied to reduce the parameter space of the measurements. We show that measurements obtained from our approach are correlated with measurements obtained from the extended Tofts model control. These correlations demonstrate that the proposed approach contains similar information to an established compartmental model and may be useful in providing an alternative theoretical framework for parameterizing mathematical models for treatment planning and diagnostic studies involving nanomedicine where convection dominated effects are important.

## 1. Introduction

The unique physiochemical and optical properties of nanoparticles make them ideal candidates for numerous biomedical applications including drug delivery, bioimaging, tissue engineering, and biosensors. Factors that influence the tumoral accumulation of nanoparticles remain an active area of interest [1,2]. Many delivery strategies for particulate systems have relied on the enhanced permeability and retention (EPR) effect for particles to passively cross the tumor endothelial barrier [3]. A common approach to improve the delivery of nanoparticles into the target site is by prolonging the retention time in the blood [4,5]. Relatively smaller size nanoparticles (<10 nm) are cleared by renal excretion directly and very large particles (>100 nm) are absorbed by liver cells [6]. Nanoparticles between 10 and 100 nm can pass through the endothelial fenestrae in the liver and spleen and may reach the target organ *via* the blood vasculature. The relationship between nanoparticle circulation time and retention in tumors is complex [7]. Systemic delivery of nanoparticle accumulation in a target tumor results from both passive transport within the convection dominated effects of blood flow within the large vasculature as well as the active biological selection and intra-endothelial transport once the particles are near the target. Recent studies have demonstrated that passive transport through inter-endothelial gaps is not responsible for the transport of nanoparticles into solid tumors [8]. Macrophages [9], as well as dendritic cells, neutrophils, and monocytes [1], have a significant role in the nanoparticle accumulation in tumors. This manuscript presents a pharmacokinetic model of nanoparticle transport within the scope of passive transport.

In particular, the pharmacokinetic model presented views the nanoparticle delivery problem as convective transport through a porous media. Imaging provides anatomical information that drives the clinical workflow and provides a natural setting for model developments and practical application. Here, model developments are with respect to computed tomography (CT). CT imaging is a widely accepted tool for use in a variety of medical imaging applications. Compared to other common imaging modalities such as magnetic resonance imaging (MRI), ultrasound (US), and positron emission tomography (PET), CT is regarded as being accessible, inexpensive, and well-tolerated by imaged subjects. The high spatial and temporal resolution makes CT a common first-line imaging modality for diagnosis and staging of primary and metastatic tumors, anticancer therapy prediction and delivery, and monitoring recurrence following therapy [10,11]. The wide availability of standardized imaging protocols enables reproducible results between scanners and institutions. In CT imaging, ionizing radiation is used to penetrate tissue with inherent contrast arising from differences in attenuation specific to each tissue type. This attenuation, expressed in Hounsfield units (HU), can be artificially increased with the addition of high-density contrast material. Measured HU values are directly proportional to the concentration of contrast in the defined area and provide the basis for quantitative CT applications [12]. While CT imaging can be performed with or without contrast, the use of contrast enables quantitative dynamic and functional imaging when used with techniques such as CT perfusion imaging. Tracer kinetic models of imaging contrast motivate our modeling approach. Contrast enhancement observed during CT perfusion imaging is used as a surrogate for tracking nanoparticle transport within this manuscript. Imaging measurements provide the data to calibrate our mathematical formulation.

High fidelity mathematical modeling of nanoparticle transport kinetics involves multiphysics fluid flow within large vessels and porous living tissue. Common mathematical formulations [13–17] consider the uptake and exchange of nanoparticles among major anatomical compartments including the liver, blood plasma, and tumor. For example, the plasma compartment represents particles in circulation and available to bind to the tumor. The liver compartment represents particles trapped by the liver to be excreted. Analysis of nanoparticle kinetics is commonly developed from first principle mass transport theory and results in a system of nonlinear ordinary differential equations describing the time-varying kinetics of the nanoparticle biodistribution. Such models incorporate several biological parameters that necessitate systematically-designed experimental data to calibrate these model parameters.

The widely applied Tofts model is a compartmental model that was designed for tissues with negligible blood volume [18]. Tofts models assume equilibrium of contrast media between the blood plasma and the extravascular-extracellular space (EES) as well as isodirectional permeability [19]. The notion of permeability in the Tofts’ approach is interpreted as the rate transfer constant between a blood vessel and the EES. The feeding vessels within the tissue are assumed to provide a spatially homogeneous arterial input function source term to the governing ordinary differential equation. Implicitly, this assumes that the time scale for the transport between imaging visible vessels and vessels not visible on imaging is less than the sequential time between a dynamic imaging acquisition. The extended Tofts models build upon the Tofts model and include additional parameters for intra-vascular signal contributions. However, permeability is still interpreted as the rate transfer constant between a blood vessel and the EES. Fundamentally, the governing equations for compartmental mass balance represent a spatially invariant mass transport between compartments and do not capture spatially variant convection phenomena.

In this manuscript, we view the transport problem as convective flow through porous tissue. While Darcy flow models have been applied to model mass transport in porous biological tissues [20–27], the key idea of this manuscript is to provide a framework for directly measuring permeability in the sense of Darcy flow through porous tissue. In the case where the porous media does not provide an imaging signal, NMR measurements may be applied to directly measure permeability and porosity [28] from an MR visible fluid. Here, the porous biological tissue provides an imaging signal. Obtaining permeability in the sense of Darcy flow is important for applications of nanotechnology in which the governing equations are dominated by convective transport [29,30]. Permeability measurements within the presented framework are obtained from the same convection dominated equations. Dimensional analysis of the parameters obtained are directly related to units of flow rates, pressure gradients, permeability, and, thus, have direct meaning for parameterizing mathematical models for treatment planning and diagnostic studies. Pixels parameter recovery results in high dimensional inverse problems that are computationally expensive [31]. An iterative clustering algorithm is applied pixel-wise and evaluated for data reduction [32].

## 2. Methods

### 2.1. Signal model

A mathematical formulation for mass transport within vascularized porous living tissue shares much in common with the mathematics, aerospace, biomedical, geosciences, and petroleum engineering mixture theory literature [33–36]. Mixture theory provides a natural framework to consider nanoparticle transport through porous tissue. At each point in space, *x* ∈ Ω, we consider the mixture of porous tissue, φ_tissue_, and blood that does, φ_nano_, and does not, φ_blood_, contain the nanoparticles:

(1) $$\varphi_{\text{tissue }}+\varphi_{\text{nano }}+\varphi_{\text{blood }}=1\;\forall x\in \Omega .$$

Mass is conserved for each component in the mixture with and without nanoparticles flowing with velocity, $v\left[\frac{m}{s}\right]$:

$$\frac{\partial \rho_{\iota }\varphi_{\iota }}{\partial t}+\nabla \cdot \left(\rho_{\iota }\varphi_{\iota }v\right)=0\;\iota \in \left\{\text{nano, blood}\right\}.$$

Mass density of each component is denoted $\rho_{\iota }\left[\frac{kg}{m^{3}}\right]$. We rewrite the mass balance in terms of the saturation:

(2) $$\frac{\partial s_{\iota }}{\partial t}+\nabla \cdot \left(s_{\iota }v\right)=0\;\iota \in \left\{\text{nano, blood}\right\},$$

(3) $$s_{\text{nano}}+s_{\text{blood}}=1\;\varphi_{\text{tissue}}=1-\phi \;\varphi_{\text{nano}}\;=\phi s_{\text{nano}}\;\varphi_{\text{blood}}=\phi s_{\text{blood}},$$

where ϕ represents the tissue porosity or void space. Saturation of a given constituent, s_1_, ι ∈ {blood, nano}, represents the ratio of the void volume filled with the constituent to the total of the void volume in the porous medium. Summing the mass balance (2) for each constituent, ι ∈ {nano, blood}, and applying the constraint that the saturations sum to one,(3), the flow is seen to be incompressible:

(4) ∇⋅v=0.

Characteristic lines of convective transport are assumed in the direction of the normal to imaging visible vessels, *n*, that are the source of the flow. The speed of the flow along the characteristic lines is denoted *a*. The governing equation for tracking nanoparticle transport is a first order hyperbolic equation:

$$\begin{array}{ll} \frac{\partial s_{\text{nano}}}{\partial t}+\frac{\partial s_{\text{nano}}}{\partial n}a=0 & a=n\cdot v=\|v\|\\ s_{\text{nano}}(0,x)=s_{0}(x) & s_{\text{nano}}(t,0)=s_{\text{AIF}}(t) \end{array}.$$

Here, the initial condition *s*_0_ may be obtained directly from the imaging data and is the only source of nanoparticle transport. The inlet boundary condition from the arterial input function, s_AIF_, is obtained by placing a region of interest (ROI) on the contrast enhancement observed in the aorta. The vector pointing from the vessel centerline implicitly defines the normal vector, *n*, from the centerline point of the closest feeding vessel, *x*_0_:

$$n=\frac{x-x_{0}}{\left\|x-x_{0}\right\|}.$$

A signed distance map [37] is used to estimate the closest perpendicular distance to the vessel input. This perpendicular distance approximation implicitly assumes that both imaging visible and non-visible vessels contribute to the flow and that vessel branches exist that are perpendicular to the imaging visible vessels. The feeding vessels within the tissue are assumed to supply the nanoparticle system input as initial conditions and boundary conditions for our system. Under these assumptions, the solution is analytic and of the form *s*(*x*, *t*) = *f*(*x*−*at*). The function, *f*, is arbitrary and is determined by the initial conditions and boundary conditions:

(5) $$s_{\text{nano}}(t,x)=s_{0}(x-at)=s_{\text{AlF }}(-x/a+t).$$

The flow speed, *a*[*m/s*], is obtained as the distance value, ||*x*–*x*_0_||, divided by the contrast bolus arrival time (BAT). The bolus arrival time is computed from the peak-gradient method available in the 3 D Slicer PkModeling extension [38]. Flow speed is assumed piecewise constant to allow for tissue heterogeneity:

(6) $$a(x)=\underset{i}{\sum }\alpha_{i}\psi_{i}(x).$$

A superpixel model [32] is used to obtain piecewise constant imaging regions. A linear iterative clustering algorithm with a grid size of 20 voxels is used to generate the super-pixel model.

### 2.2. Flow model

Following Darcy’s law, the flow speed *a* is the assumed result of a pressure gradient along the flow direction:

(7) $$v\cdot n=-\frac{\kappa }{\mu }\nabla p\cdot n=a.$$

The flow velocity, *v*, is proportional to the pressure gradient, *p*, across the tissue and in the direction of the normal, *n*, from the source vessel. Tissue permeability is denoted κ[*m*^2^]. The viscosity of blood is known, μ = 3.5 · 10^−3^ [*Pa* · *s*]. Poiseuille’s law [39] provides a relationship between the flow rates and pressure gradient in the vessels:

(8) $$Q=\frac{\pi R^{4}}{8\mu }\nabla p\cdot n.$$

Flow rates, *Q*, in rabbit liver have been measured as 177[*ml/min*] [40]. The average vessel radius, *R*, was extracted from image Hessian based filters applied to the image subtraction of the pre-contrast image from the maximum intensity projection image [39]. The pressure gradient is *assumed* spatially homogeneous across the tissue and vessels. Substituting Equation (8) into Equation (7) provides an estimate of the tissue permeability:

(9) $$\kappa =\frac{a\pi R^{4}}{8Q}.$$

Within this manuscript, Equation (9) estimates the permeability as proportional to the flow speed. However, for further comparison with existing literature, it is worth noting that alternative measurements of the flow rate, *Q*, may be obtained from indicator dilution theory [41] and blood volume measurements [42]. Flow rates are derived from the conservation of flow across the tissue and vessel interface. The flow rate input is equal to the flow rate out of the tissue. A constant velocity over each interface, ∂Ω, is assumed. The flow rate, *Q* is derived from the integral form of mass conservation applied to constituent ι at the tissue interface:

(10) $$m_{\iota }(T)-m_{\iota }(0)=\int_{\Omega }\rho \varphi (x,T)dV-\int_{\Omega }\rho \varphi (x,0)dV\;=\int_{0}^{T}\int_{\partial \Omega }\rho \varphi v\cdot n\;\iota \in \left\{\text{nano, blood}\right\}.$$

Under the assumption that the time interval *t* = 0 represents the time prior to the nanoparticle arrival and *t* = *T* is less than the transit time out of the tissue Ω, outflow is not considered. Within the time interval [0, *T*], the flow rate into the tissue may be obtained:

(11) $$m_{\text{nano}}(T)=\int_{0}^{T}\rho \varphi_{\text{nano}}dt\int_{\partial \Omega }v_{\text{in}}\cdot ndA\;\Rightarrow \;Q\;=\int_{\partial \Omega }v_{\text{in}}\cdot ndA=\frac{m(T)}{\int_{0}^{T}\rho \varphi_{\text{nano}}dt}.$$

Here, steady state input velocity, *v*_in_, is assumed so that the velocity may be factored out of our the integral in time. The tracer distribution is assumed homogeneous across the feeding vessel such that the concentration factors out of the spatial integrals. At this step, the various analysis methods depart in assumptions for calculation of the flow rate, *Q*. Indicator dilution theory [41] assumes that the mass in the tissue is known from the injected concentration bolus, *m*(*T*) = *m*_0_. Calculations of blood volume (BV) measurements use the area under the curve for the concentration in the tissue [42]. Dimensional analysis of the units of each term are used to guide the algebraic relationships seen in the literature. The average flow rate, *Q*, may be related to BV as

(12) $$Q\equiv \frac{1}{\Delta t}\int_{0}^{T}Q=\frac{1}{\Delta t}\int_{\Omega }BV(x)dV\;BV(x)\equiv \frac{\int_{0}^{T}\varphi_{\text{nano}}(x,t)dt}{\int_{0}^{T}\rho \varphi_{\text{nano}}dt}.$$

### 2.3. Compartmental model

In the assessment of tumor biology and angiogenesis, compartmental analysis of CT perfusion imaging provides a noninvasive volumetric assessment of tumor vasculature [11,43–46]. Correlation between parametric maps obtained from compartmental analysis and microvessel structure has been used as a predictor of disease severity and treatment outcomes [47–50].

We apply the PkModeling extension for 3 D Slicer [51] to compute the extended Tofts compartmental model parameters as a control for our speed recovery in Equation (5):

(13) $$s_{\text{nano }}(t)=K^{\text{trans}}\int_{0}^{t}S_{\text{aif }}(u)e^{-\frac{K^{\text{trans}}}{v_{e}}(t-u)}du+fpvs_{\text{aif}}(t).$$

The solution to the compartmental model was obtained from a nonlinear least square curve fit to the extended Tofts model analytical solution parametrized by *K*_trans_, *v*_*e*_, and *fpv*. Here, K_trans_[1/*s*] represents the influx forward volume transfer constant (into EES from plasma), *v*_*e*_ is the fractional volume of EES per unit volume of tissue, *fpv* represents the fractional plasma volume of the arterial input present at each voxel [19]. Contrast enhancement is linearly proportional to the concentration of contrast agent in the tissue [42]:

(14) $$\varphi_{\text{nano}}\propto I(x,t)-I_{b}(x),$$

where*l*_*b*_(*x*) represents a baseline value of the tissue before contrast enhancement. The correlation between the Tofts model parameters and speed a estimates are measured.

### 2.4. Experimental methods

Animal procedures were performed following a protocol approved by the MD Anderson Cancer Center Institutional Animal Care and Use Committee per recommendations in the National Institutes of Health Guide for Care and Use of Laboratory Animals. Four male New Zealand White rabbits (Charles River Laboratories, Massachusetts, USA) weighing 2.4–3 kg underwent VX2 tumor inoculation in the left lateral lobe of the liver. The four animals are identified as id1, id2, id3, and id4 in our analysis. Tumor implantation was performed *via* the administration of freshly harvested and prepared VX2 tumor fragments from an in-house VX2 donor line. VX2 fragments 3–4 mm in diameter were implanted in the liver at a single site through an 18 GA needle attached to a 1 ml syringe. Tumors were allowed to grow for 1014 days until the tumors reached approximately 1 cm diameter. Following sufficient tumor growth, all animals underwent a sham procedure involving the insertion and removal of a catheter (20 GA × 1.88 in). CT imaging occurred at the time of the sham procedure and 24 h post-procedure.

Before imaging, rabbits were anesthetized with isoflurane (1–5%)/oxygen (1.5L/min) administered *via* endotracheal tube and laryngeal mask. CT imaging was performed using a multislice detector CT (SOMATOM Definition Edge, Siemens Healthineers, Erlangen, Germany) to determine contrast transport through the liver and tumor. A vac fix immobilization device was used to maintain supine positioning on the scanner table. Perfusion imaging was acquired with 4, 6, or 8 ml of 320 mg/ml iodixanol contrast (Visipaque, GE Healthcare, Cork, Ireland) injected at a rate of at 2 ml/sec into the marginal ear vein using a power injector (Medrad Envision, CT, Connecticut, USA). Scan parameters were 80 kVp, 264 mA, 570 ms rotation time, and pitch of 0.5. Scans were acquired over the entire liver before contrast injection began, and at delays of 2, 4, and 6 s following the injection start; the total scanning time was 87 s. A 15 cm field-of-view was reconstructed from the raw projection data with a slice thickness of 1.5 mm and an interval of 1.0 mm with a B20f reconstruction kernel.

## 3. Results

**Figure 1.**
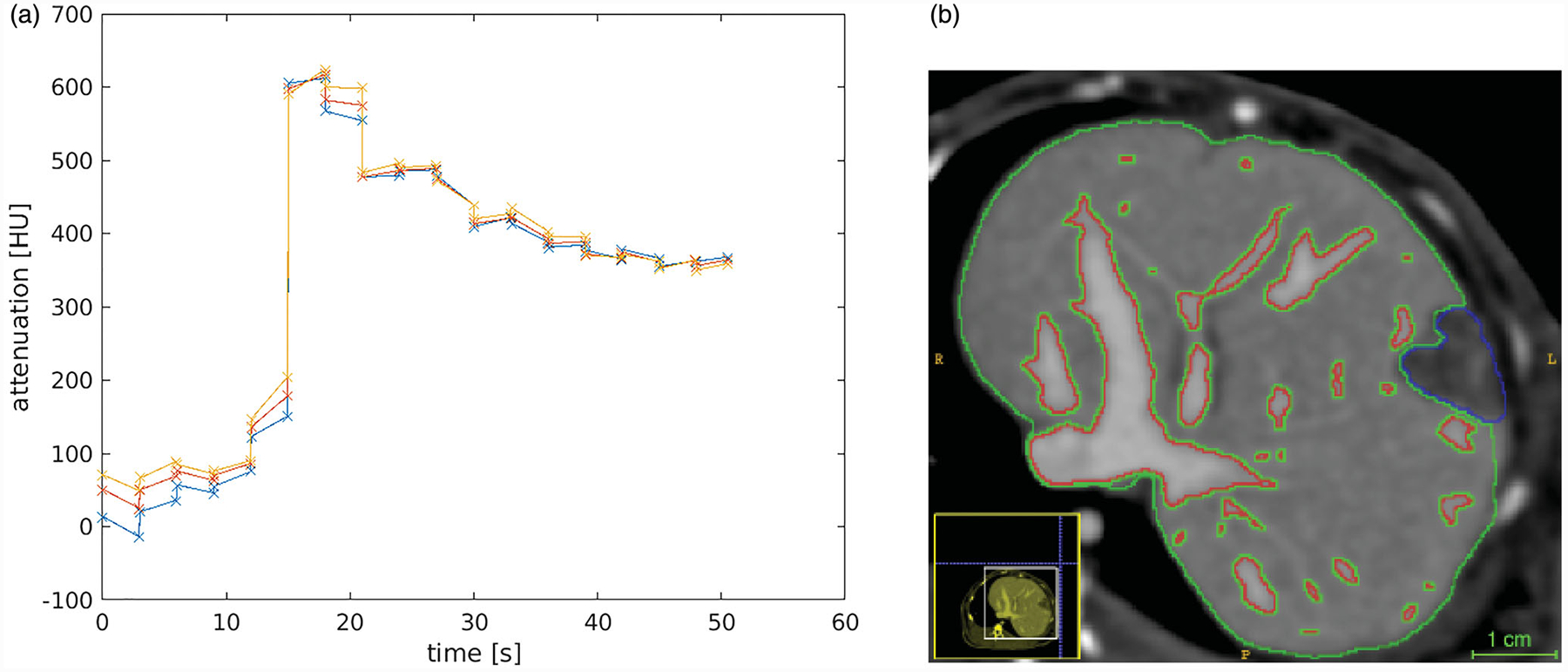
*In vivo* data. (a) The time history of the contrast in the aorta illustrates the bolus of contrast arrival time. The different lines illustrate three representative pixels. (b) The labeled regions illustrate the imaging visible vessels (red), the tumor (blue), and the liver parenchyma tissue (green). Pixelwise distance from the nearest vessel boundary provides the spatial length scale for our governing hyperbolic equation for convective transport.

Transport of iodinated contrast agent is used as a surrogate for nanoparticle transport in the tissue. Datum used in the *in vivo* evaluation of our model is shown in Figure 1. Figure 1(a) illustrates the time history of the contrast uptake at the aorta used for as the arterial input, s_AIF_. Regions of interest used in the data analysis are outlined in Figure 1(b). Imaging visible vessels are outlined in red. Pixel-wise distance is measured from the perpendicular distance of the closest vessel outlined. The blue region outlines the VX2 tumor growth. Green outlines liver parenchyma tissue.

**Figure 2.**
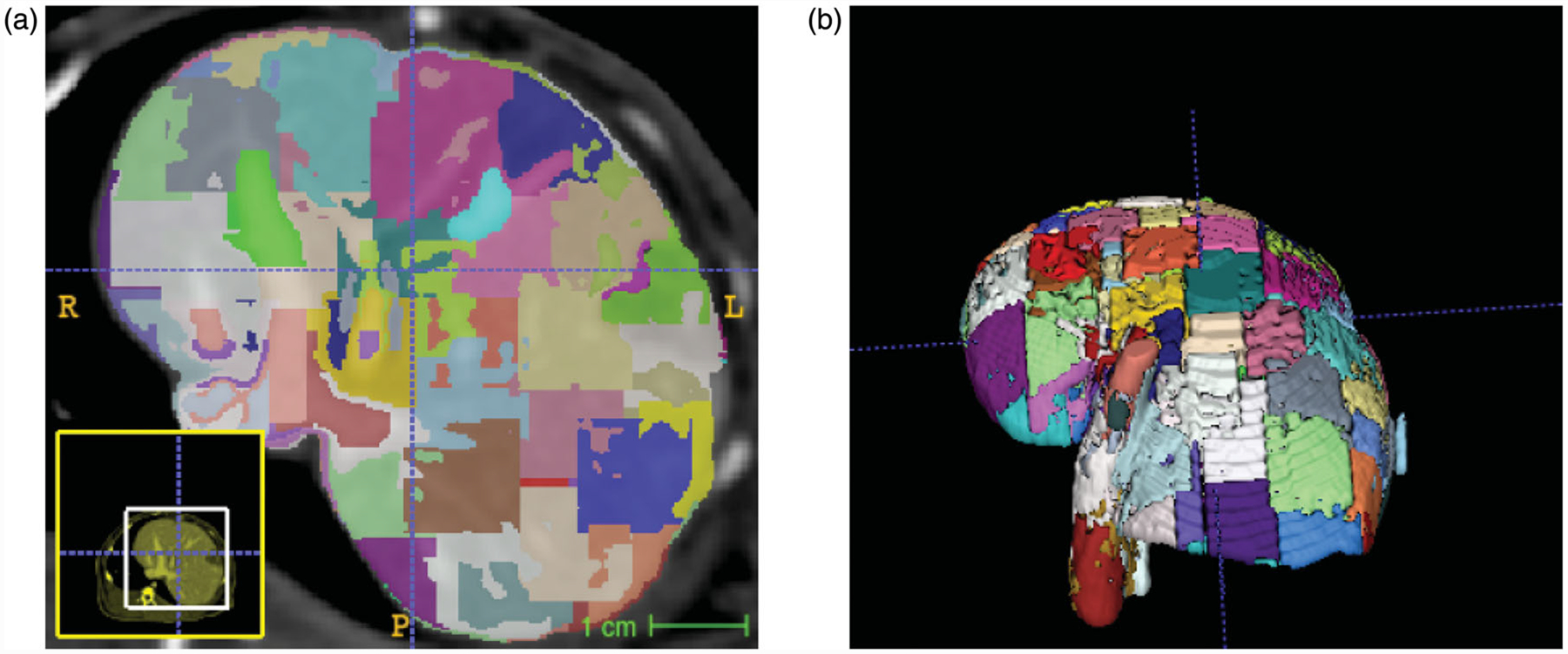
Domain decomposition of liver anatomy with simple linear iterative clustering (SLIC) segmentation. An (a) axial view and (b) 3 D visualization of the domain decomposition is shown.

Linear iterative clustering segmentation of the rabbit liver resulted in 174, 176, 111, and 156 super-pixel regions within the livers of animal id1, id2, id3, and id4, respectively. Two iterations of iterative clustering were performed in each data set for convergence. The average volume of each superpixel was 960 ±26 mm^3^. A representative example of the pixelation of the liver is shown in Figure 2. Super-pixel regions form the piecewise constant basis for inversion of the tissue heterogeneity.

**Figure 3.**
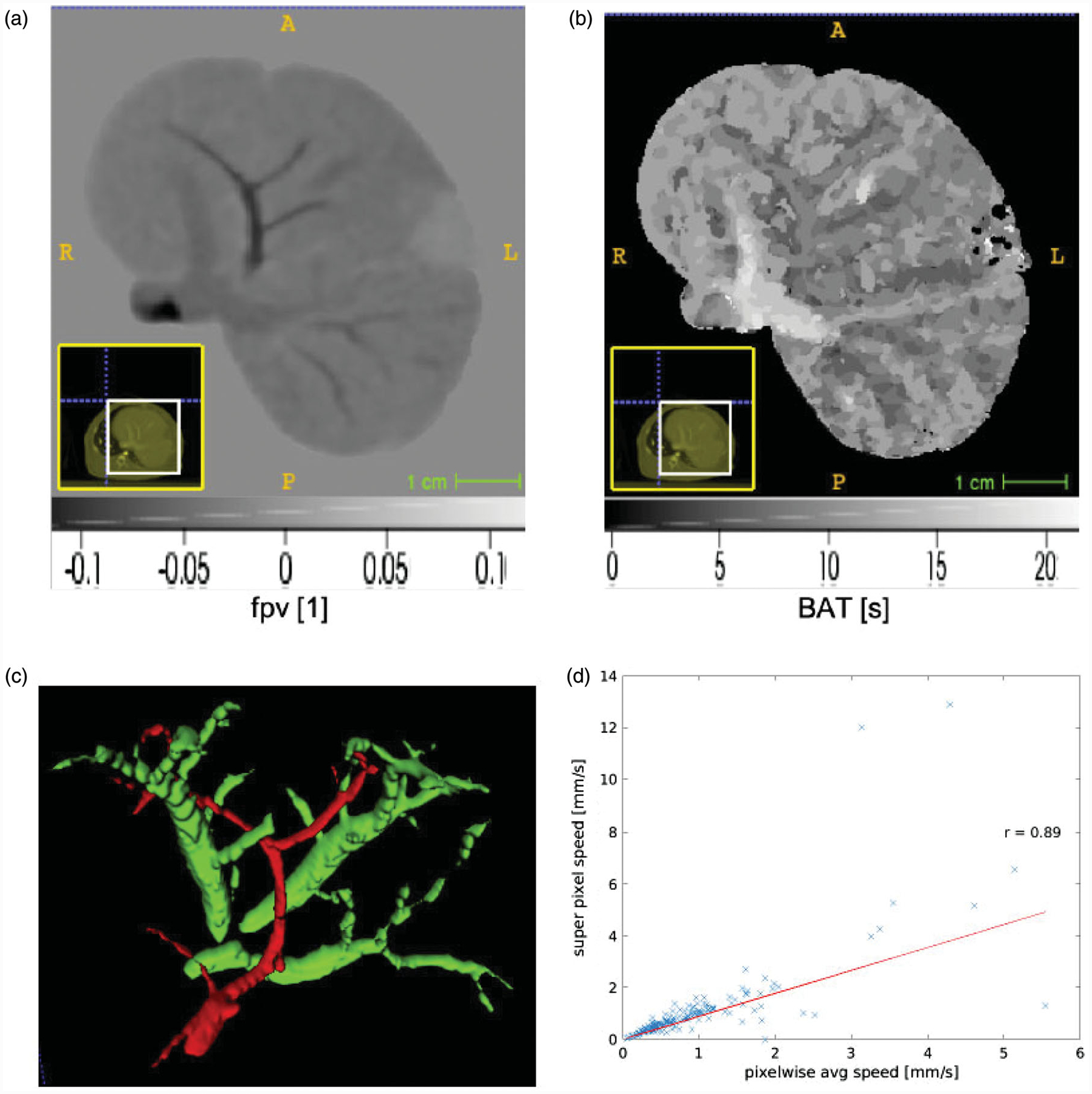
An overview of our algorithmic approach is shown for animal id1. (a) The extended Tofts model provides a control for the proposed approach. Spatial variations in the tissue fractional plasma volume parameter *fpv* within the parenchyma and vessels is shown. Negative values are the result of the curve fit of the analytical model in Equation (13) to the time-intensity data at each pixel. (b) Spatial variations in the bolus arrival time is measured in seconds. (c) Distance information is computed with respected to imaging visible vessels. An example of the 3 D vessel segmentation used to calculate the signed distance transform *x*−*x*_0_ is shown. (d) The bolus arrival time combined with the distance information provide estimates of the flow speed. Correlation between the super pixel recovered speed and the average of the pixelwise is shown. Within these pixels the average speed represented by the super pixel is expected to provide dimension reduction of the parameter optimization space while maintaining accuracy of the solution field.

Figure 3 provides a visualization the data input to our algorithm as well as a visualization of the extended Tofts model results. Figure 3(a) illustrates spatial variations in the fractional plasma volume, *fpv*. Fractional plasma volume is a dimensionless quantity [1]. Negative values of the fractional plasma volume are seen in the curve fit of the extended Tofts model (13) to the data. Figure 3(b) illustrates the pixel-wise bolus arrival time in seconds. Voxel neighborhoods of pixels are seen to have the same arrival time. Figure 3(c) illustrates surface of the imaging visible vessels. Flow speed is estimated as the distance to the nearest vessel surface divided by the arrival time. Correlations between the extend Tofts model parameters and our measurement flow speed are summarized in Table 1. The fractional plasma volume, *fpv*, extended Tofts parameter shows a positive correlation with our flow speed measurements for all animals.

**Figure 4.**
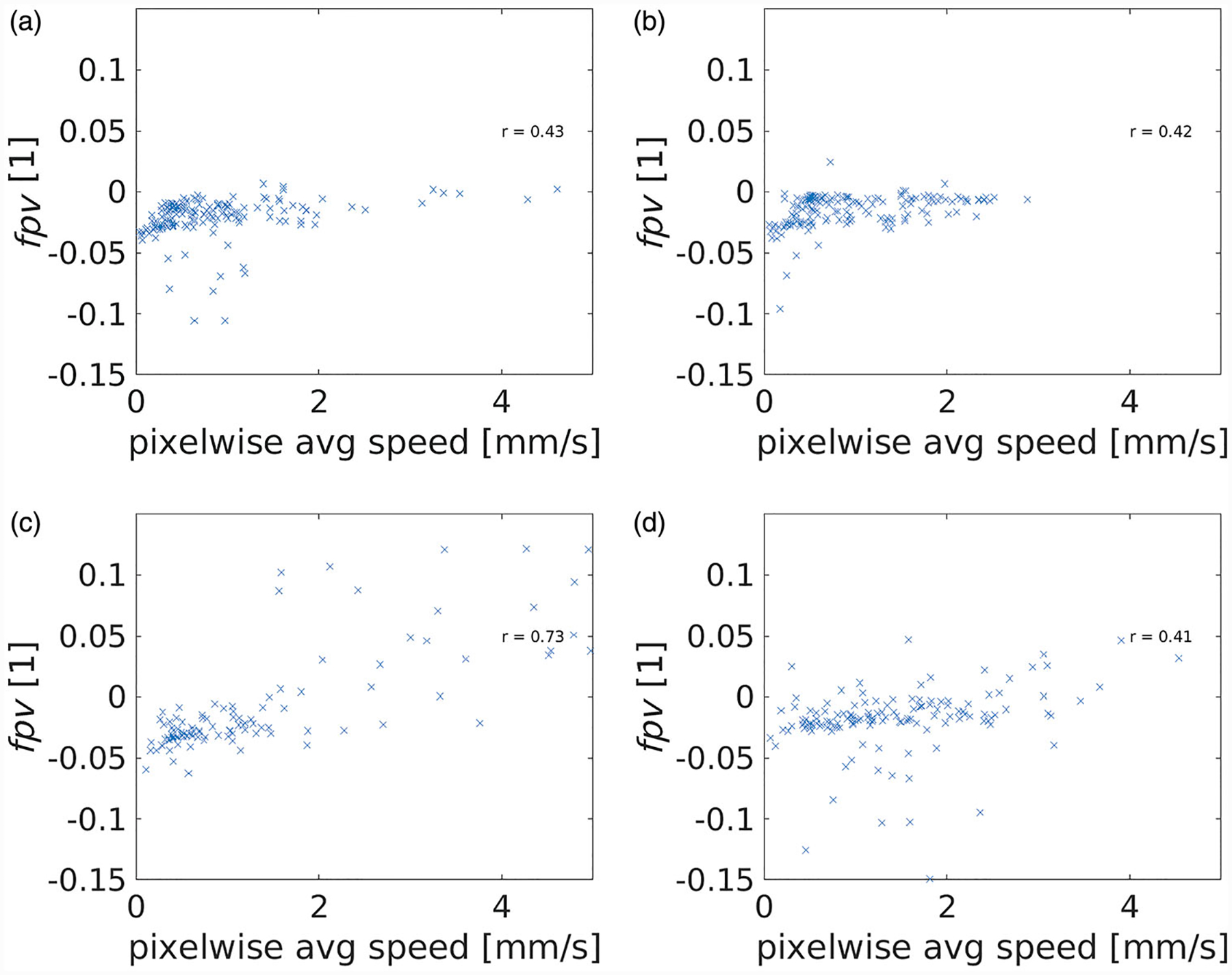
Tofts model, *fpv*, versus flow speed estimates are provided. The fractional plasma volume is a dimensionless [1] parameter. Animal id1, id2, id3, and id4 are shown in (a), (b), (c), and (d); respectively. The correlation, *r*, between the fractional plasma volume and flow speed is provided within each dataset.

Figure 3(d) plots the average pixel flow speed against the flows speed recovered directly from superpixel domain decomposition for animal id1. The x-axis represents the pixel-wise speed averaged across a given super pixel illustrated in Figure 2. The y-axis represents the speed recovered from our model fits applied to the raw data averaged across a super-pixel. A Pearson correlation of 0.95 (*p* <.05) was observed and indicates that the superpixel domain decomposition is a good approximation of the tissue heterogeneity. Table 2 provides a global summary of the pixelwise average permeability estimate with each animal liver, id1, id2, id3, and id4. Permeability was calculated according to Equation (9). The average vessel radius extracted from the surface of the imaging visible vessels shown in Figure 3(c) was 1.09mm, 1.07mm, 1.10mm, and 1.09mm for animal id1, id2, id3, and id4; respectively. Figure 4 provides a comprehensive quantitative summary of the correlations measured between the pixelwise average speed and the extended Tofts *fpv* parameter

## 4. Discussion

Families of tracer kinetic models may be grouped as model-independent based on Fick’s Law, deconvolution analysis, compartmental modeling, and modeling that accounts for convective transport [42]. Each has assumptions appropriate for domain-specific applications. Here we assume that convective transport from the vessels directly supplies the flow within the tissue. Importantly, our approach allows us to recover permeability properties with respect to porous media flow. Table 2 presents initial steps and demonstrates the feasibility in the recovery of convection related properties from imaging data. As a reference, permeability measurements on the order of 1*e*^−7^*m*^2^ are similar to gravel or fractured rock within the oil and gas communities [52]. Tofts model proposes a spatially invariant compartmental formulation. The resulting measurements of the Tofts formulation have different units, [1/s] for *K*_trans_ and dimensionless for *v*_*e*_ and *fpv*, with respect to transport equations within porous tissue. Calibrating and validation of the same governing equations have the benefit of direct interpretability. Although our approach applied the model to contrast-enhanced imaging, imaging visible measurements of nanoparticle concentrations may be used to directly recover permeability measurements of interest for transport within porous tissue.

This manuscript presents a pharmacokinetic model of nanoparticle transport within the scope of passive transport. Permeability estimates (Table 2) are with respect to contrast flow through porous tissue. Antibody targeting and interactions with the immune system [1] are not considered in this approach. However, the key ideas of the framework may also be coupled with mechanisms for active transport of nanoparticles into the target tumor. This may be modeled as a sink term with appropriate rate constants within our governing equations. For example, within a mixture theory framework, the concentration of immune cells may be included as an additional constituent within Equation (1). Rate constants for the uptake of the nanoparticles by the immune system formulates the interaction as a first-order relationship between the presence of nanoparticles and uptake by the immune system. Additional model complexity may incorporate nanoparticle interactions with blood cells, plasma proteins and complement proteins. Serum proteins present in the bloodstream bind to the nanoparticles and results in their rapid recognition and uptake of nanoparticles by the mononuclear phagocyte system. Premature clearance of nanoparticles by the mononuclear phagocyte system due to their size and nonspecific binding limit the delivery efficacy of nanoparticles into a specific target site [4]. Additional constituents couples additional equations and parameters to our governing equations. Solution of the governing equations coupled with immune cells share much in common within the field of tumor growth modeling [53]. These models are based on the conservation laws of continuum physics complemented by source and flux characterizations that trigger and control cancer development and decline through mathematical representations of the events listed in the Hallmarks of Cancer [54].

The presented methodology is applicable to flow rates characteristic of systemic delivery as well as convection-enhanced delivery. Convection enhanced delivery applied to nanomedicine utilizes direct injections of the nanotechnology into the interstitial space to achieve the desired therapeutic or diagnostic effect. Planning software for convection-enhanced delivery is likely to be useful in guiding in deciding catheter placement [26]. Our parameter recovery approach provides a methodology to recover patient-specific transport parameters with direct meaning with respect to convection-dominated governing equations. Either a literature value for pressure may be used to recover the permeability, or the framework may be applied with patient-specific blood pressure measurements. The direct correlation between flow speed and permeability is expected to provide a reliable reproducible methodology for Darcy’s Law based predictions.

Limitations in these measurements arise from the tradeoffs in computational efficiency and model fidelity. The analytical solution in Equation (5) facilitates an efficient numerical solution but is inherently one-dimensional from the closest perpendicular distance to the nearest vessel source. Sufficiently near a vessel source, this approximation is likely to be appropriate. Future efforts will evaluate tradeoff of increase model fidelity. Finite element methods are needed to curve fit the 4 D enhancement data to the partial differential equations for mass balance, Equation (2), coupled with incompressible flow, Equation (4). The finite element method further requires a computation mesh that accurately conforms the tissue anatomy. Adjoint methods [31] are needed to provide analytic gradients with respect to the domain decomposition for efficient model calibration to the data. The presented results are a theoretically sound simplification of the 4 D partial differential equation constrained optimization using a finite element approach. Here, we hypothesize that any solution bias induced by our simplified approach may be reconciled with appropriate calibration and cross-validation to imaging data. Thus, the overall correlation between the recovered permeability and permeability of a high fidelity finite element approach is anticipated to be high but with much less cost.

Future work will also incorporate numerical simulations of the flow through the vessels into the transport problem. The problem of particle flow through the vasculature has been considered for varying particle diameters. Navier-Stokes computational fluid dynamic simulations are considered for flow through the vasculature. For large particle size relative to the vessels, perturbations of the fluid flow from the presence of the particles should be considered. However, for nanomedicine, the particles are small relative to capillary diameters (≈ 10 μm). The fluid force on the particle should be considered, however, the inverse particle momentum exchange is not considered. This one-way coupling approach has been shown to save significant computational resources while providing accurate results in this small nanoparticle to vessel diameter setting [55]. For systemic delivery, tracking of flow streamlines from the fluid flow simulations provides a mechanism for downstream targeting of the desired delivery [56]. Runge-Kutta methods are used to solve the differential equations with a varying time step. Ultimately, for a given application and data quality, multiple models may be considered feasible to describe the data. Future efforts will apply Bayesian model selection approaches to a family of models to balance model complexity with the goodness of fit [18].

## Figures and Tables

**Table 1.** Summary of extended Tofts analysis.

	id1	id2	id3	id4
*r*(*a, K*_trans_)	−0.27	−0.32	0.35	−0.05
*r*(*a, v*_*e*_)	−0.16	−0.30	0.23	−0.38
*r*(*a, fpv*)	0.43	0.42	0.73	0.41

The observed correlation, *r*, between the measured flow speed (6) and the *K*_trans_, *v*_*e*_, *fpv*, extended Tofts model parameters (13) are shown for each animal: id1, id2, id3, and id4.

**Table 2.** Summary of permeability measurements.

	id1	id2	id3	id4
κ[*m*^2^]	1.74e–07	1.72e–07	2.83e–07	2.79e–07

The pixelwise average permeability estimates with the liver parenchyma tissue is shown for each animal: id1, id2, id3, and id4.

## References

[1] Korangath P, Barnett JD, Sharma A, Nanoparticle interactions with immune cells dominate tumor retention and induce t cell-mediated tumor suppression in models of breast cancer. Sci Adv. 2020;6(13):eaay1601. 32232146 10.1126/sciadv.aay1601 PMC7096167

[2] Ernsting MJ, Murakami M, Roy A, Factors controlling the pharmacokinetics, biodistribution and intratumoral penetration of nanoparticles. J Control Release. 2013;172(3):782–794. 24075927 10.1016/j.jconrel.2013.09.013 PMC3891171

[3] Decuzzi P, Pasqualini R, Arap W, Intravascular delivery of particulate systems: does geometry really matter? Pharm Res. 2009;26(1):235–243. 18712584 10.1007/s11095-008-9697-x

[4] Gustafson HH, Holt-Casper D, Grainger DW, Nanoparticle uptake: the phagocyte problem. Nano Today. 2015;10(4):487–510. 26640510 10.1016/j.nantod.2015.06.006 PMC4666556

[5] Kievit FM, Zhang M. Cancer nanotheranostics: improving imaging and therapy by targeted delivery across biological barriers. Adv Mater. 2011;23(36):H217–H247. 21842473 10.1002/adma.201102313 PMC3397249

[6] Chithrani BD, Ghazani AA, Chan WCW. Determining the size and shape dependence of gold nanoparticle uptake into mammalian cells. Nano Lett. 2006;6(4):662–668. 16608261 10.1021/nl052396o

[7] Soetaert F, Korangath P, Serantes D, Cancer therapy with iron oxide nanoparticles: Agents of thermal and immune therapies. Adv Drug Delivery Rev. 2020. doi:10.1016/j.addr.2020.06.025 PMC7736167 32603814

[8] Sindhwani S, Syed AM, Ngai J, The entry of nanoparticles into solid tumours. Nat Mater. 2020;19(5):566–575. 31932672 10.1038/s41563-019-0566-2

[9] Liu LY, Ma XZ, Ouyang B, Nanoparticle uptake in a spontaneous and immunocompetent Woodchuck Liver Cancer Model. ACS Nano. 2020;14(4):4698–4715. 32255624 10.1021/acsnano.0c00468

[10] Kim SH, Kamaya A, Willmann JK. Ct perfusion of the liver: principles and applications in oncology. Radiology. 2014;272(2): 322–344. 25058132 10.1148/radiol.14130091 PMC4263626

[11] Ippolito D, Fior D, Bonaffini PA, Quantitative evaluation of ct-perfusion map as indicator of tumor response to transarterial chemoembolization and radiofrequency ablation in hcc patients. Eur J Radiol. 2014;83(9):1665–1671. 24962900 10.1016/j.ejrad.2014.05.040

[12] Axel L Cerebral blood flow determination by rapid-sequence computed tomography: theoretical analysis. Radiology. 1980; 137(3):679–686. 7003648 10.1148/radiology.137.3.7003648

[13] Bischoff KB, Dedrick RL, Zaharko DS, Methotrexate pharmacokinetics. J Pharm Sci. 1971;60(8):1128–1133. 5127083 10.1002/jps.2600600803

[14] Frieboes HB, Wu M, Lowengrub J, A computational model for predicting nanoparticle accumulation in tumor vasculature. PLoS One. 2013;8(2):e56876. 23468887 10.1371/journal.pone.0056876 PMC3585411

[15] Gilkey MJ, Krishnan V, Scheetz L, Physiologically based pharmacokinetic modeling of fluorescently labeled block copolymer nanoparticles for controlled drug delivery in leukemia therapy. CPT Pharmacometrics Syst. Pharmacol 2015;4(3):167–174. 10.1002/psp4.13 PMC4394613 26225236

[16] Li M, Panagi Z, Avgoustakis K, Physiologically based pharmacokinetic modeling of plga nanoparticles with varied mpeg content. Int J Nanomedicine. 2012;7(5):1345–1356. 22419876 10.2147/IJN.S23758 PMC3299578

[17] Elgrabli D, Beaudouin R, Jbilou N, Biodistribution and clearance of TiO2 Nanoparticles in Rats after Intravenous Injection. PLoS One. 2015;10(4):e0124490. 25909957 10.1371/journal.pone.0124490 PMC4409301

[18] Duan C, Kallehauge JF, Bretthorst GL, Are complex dce-mri models supported by clinical data? Magn Reson Med. 2017;77(3): 1329–1339. 26946317 10.1002/mrm.26189 PMC5548456

[19] Chikui T, Obara M, Simonetti AW, The principal of dynamic contrast enhanced mri, the method of pharmacokinetic analysis, and its application in the head and neck region. Int J Dent. 2012; 2012:480659. 23118750 10.1155/2012/480659 PMC3483829

[20] Fuentes D, Fahrenholtz SJ, Guo C, Mathematical modeling of mass and energy transport for thermoembolization. Int J Hyperthermia. 2020;37(1):356–365. 32308071 10.1080/02656736.2020.1749317 PMC10558277

[21] Soltani M, Chen P. Numerical modeling of fluid flow in solid tumors. PloS One. 2011;6(6):e20344. 21673952 10.1371/journal.pone.0020344 PMC3108959

[22] Khaled A-RA, Vafai K. The role of porous media in modeling flow and heat transfer in biological tissues. Int J Heat Mass Transf. 2003;46(26):4989–5003.

[23] Salama A, El-Amin MF, Abbas I, On the viscous dissipation modeling of thermal fluid flow in a porous medium. Arch Appl Mech. 2011;81(12):1865–1876.

[24] Tapani E, Vehmas T, Voutilainen P. Effect of injection speed on the spread of ethanol during experimental liver ethanol injections. Acad Radiol. 1996;3(12):1025–1029. 9017018 10.1016/s1076-6332(96)80038-9

[25] Boucher Y, Brekken C, Netti PA, Intratumoral infusion of fluid: estimation of hydraulic conductivity and implications for the delivery of therapeutic agents. Br J Cancer. 1998;78(11): 1442–1448. 9836476 10.1038/bjc.1998.705 PMC2063228

[26] Magdoom KN, Pishko GL, Rice L, Mri-based computational model of heterogeneous tracer transport following local infusion into a mouse hind limb tumor. PloS One. 2014;9(3):e89594. 24619021 10.1371/journal.pone.0089594 PMC3949671

[27] Barauskas R, Gulbinas A, Barauskas G. Finite element modeling and experimental investigation of infiltration of sodium chloride solution into nonviable liver tissue. Medicina (Kaunas). 2007;43(5): 399–411. 17563417

[28] Honarpour M, Mahmood SM. Relative-permeability measurements: an overview. J Petrol Technol. 1988;40(08):963–966.

[29] Chauhan VP, Stylianopoulos T, Martin JD, Normalization of tumour blood vessels improves the delivery of nanomedicines in a size-dependent manner. Nat Nanotechnol. 2012;7(6):383–388. 22484912 10.1038/nnano.2012.45 PMC3370066

[30] Xi G, Robinson E, Mania-Farnell B, Convection-enhanced delivery of nanodiamond drug delivery platforms for intracranial tumor treatment. Nanomedicine. 2014;10(2):381–391. 23916888 10.1016/j.nano.2013.07.013

[31] Fuentes D, Elliott A, Weinberg JS, An inverse problem approach to recovery of in vivo nanoparticle concentrations from thermal image monitoring of mr-guided laser induced thermal therapy. Ann Biomed Eng. 2013;41(1):100–111. 22918665 10.1007/s10439-012-0638-9 PMC3524364

[32] Lowekamp BC, Chen DT, Yaniv Z, Scalable simple linear iterative clustering (sslic) using a generic and parallel approach. 2018. https://arxiv.org/abs/1806.08741

[33] Peaceman DW. Fundamentals of numerical reservoir simulation. New York (NY): Elsevier; 1977.

[34] Faust CR, Mercer JW. Geothermal reservoir simulation: 1. mathematical models for liquid-and vapor-dominated hydrothermal systems. Water Resour Res. 1979;15(1):23–30.

[35] Kee RJ, Coltrin ME, Glarborg P. Chemically reacting flow: theory and practice. Hoboken (NJ): John Wiley & Sons; 2005.

[36] Oden JT, Hawkins A, Prudhomme S. General diffuse-interface theories and an approach to predictive tumor growth modeling. Math Models Methods Appl Sci. 2010;20(03):477–517.

[37] Maurer CR, Qi R, Raghavan V. A linear time algorithm for computing exact euclidean distance transforms of binary images in arbitrary dimensions. IEEE Trans Pattern Anal Mach Intell. 2003;25(2): 265–270.

[38] Yingxuan Z, Andrey Fedorov SPL, John Evans MGH, Pkmodeling module–3d slicer. [cited 2015 Apr 17]. Available from: https://www.slicer.org/slicerWiki/index.php/Documentation/Nightly/Modules/PkModeling.

[39] Chengyue W, Hormuth DA, Oliver TA, Patient-specific characterization of breast cancer hemodynamics using image-guided computational fluid dynamics. IEEE Trans Med Imaging. 2020;39(9):2760–2771. 32086203 10.1109/TMI.2020.2975375 PMC7438313

[40] Davies B, Morris T. Physiological parameters in laboratory animals and humans. Pharm Res. 1993;10(7):1093–1095. 8378254 10.1023/a:1018943613122

[41] Doriot PA, Dorsaz PA, Dorsaz L, Is the indicator dilution theory really the adequate base of many blood flow measurement techniques? Med Phys. 1997;24(12):1889–1898. 9434971 10.1118/1.598102

[42] Lee T-Y. Functional ct: physiological models. Trends Biotechnol. 2002;20(8):S3–S10.12570152

[43] Brix G, Zwick S, Griebel J, Estimation of tissue perfusion by dynamic contrast-enhanced imaging: Simulation-based evaluation of the steepest slope method. Eur Radiol. 2010;20(9):2166–2175. 20407900 10.1007/s00330-010-1787-6

[44] Goetti R, Leschka S, Desbiolles L, Quantitative computed tomography liver perfusion imaging using dynamic spiral scanning with variable pitch: feasibility and initial results in patients with cancer metastases. Investig Radiol. 2010;45(7): 419–426. 20498611 10.1097/RLI.0b013e3181e1937b

[45] Miles KA, Hayball MP, Dixon AK. Functional images of hepatic perfusion obtained with dynamic ct. Radiology. 1993;188(2): 405–411. 8327686 10.1148/radiology.188.2.8327686

[46] Wang Y, Hobbs BP, Ng CS. Ct perfusion characteristics identify metastatic sites in liver. Biomed Res Int. 2015;2015:120749. 26509144 10.1155/2015/120749 PMC4609766

[47] Van Beers BE, Leconte I, Materne R, Hepatic perfusion parameters in chronic liver disease: dynamic ct measurements correlated with disease severity. Am J Roentgenol. 2001;176(3): 667–673. 11222202 10.2214/ajr.176.3.1760667

[48] Kim KA, Choi SY, Kim MU, The efficacy of cone-beam ct-based liver perfusion mapping to predict initial response of hepatocellular carcinoma to transarterial chemoembolization. J Vasc Interven Radiol. 2019;30(3):358–369. 10.1016/j.jvir.2018.10.002 30819478

[49] Goh V, Halligan S, Daley F, Colorectal tumor vascularity: quantitative assessment with multidetector ct-do tumor perfusion measurements reflect angiogenesis? Radiology. 2008;249(2): 510–517. 18812560 10.1148/radiol.2492071365

[50] Tamandl D, Waneck F, Sieghart W, Early response evaluation using ct-perfusion one day after transarterial chemoembolization for hcc predicts treatment response and long-term disease control. Eur J Radiol. 2017;90:73–80. 28583650 10.1016/j.ejrad.2017.02.032

[51] Kikinis R, Steve DP, Vosburgh KG. 3d slicer: a platform for subject-specific image analysis, visualization, and clinical support. In: Jolesz F, editor. Intraoperative imaging and image-guided therapy. New York (NY): Springer; 2014. p. 277–289.

[52] Jacob B Dynamics of fluids in porous media. New York (NY): Courier Dover Publications; 2013.

[53] Oden JT, Lima EABF, Almeida RC, Toward predictive multiscale modeling of vascular tumor growth. Arch Computat Methods Eng. 2016;23(4):735–745.

[54] Hanahan D, Weinberg RA. Hallmarks of cancer: the next generation. Cell. 2011;144(5):646–674. 21376230 10.1016/j.cell.2011.02.013

[55] Mukherjee D, Padilla J, Shadden SC. Numerical investigation of fluid-particle interactions for embolic stroke. Theor Comput Fluid Dyn. 2016;30(1–2):23–39.

[56] Basciano CA, Kleinstreuer C, Kennedy AS, Computer modeling of controlled microsphere release and targeting in a representative hepatic artery system. Ann Biomed Eng. 2010;38(5): 1862–1879. 20162358 10.1007/s10439-010-9955-z

